# Protein Self-Interaction in Cellular Function and Network Evolution: Molecular Mechanisms, AI-Driven Insights, and Therapeutic Potentials

**DOI:** 10.3390/ijms27156776

**Published:** 2026-07-29

**Authors:** Yuanxiao Gao, Wenyu Zhang, Guang Hu

**Affiliations:** 1 School of Mathematics and Data Science, Shaanxi University of Science and Technology, Xi’an 710021, China; 2 School of Life Science and Technology, Northwestern Polytechnical University, Xi’an 710129, China; wyzhang@nwpu.edu.cn; 3 Biomedical Basic Research Center (BBRC) of Jiangsu, MOE Key Laboratory of Geriatric Diseases and Immunology, School of Life Sciences, Soochow University, Suzhou 215123, China; 4 Institute of Health Data Science at Soochow University, Suzhou 215123, China

**Keywords:** protein self-interaction, homo-oligomerization, deep learning, AlphaFold, liquid–liquid phase separation, allosteric regulation, protein aggregation, neurodegenerative disease

## Abstract

Protein self-interaction to form homodimers and higher-order homo-oligomers is a ubiquitous phenomenon fundamental to living organisms. Recent structural, system-level, and computational insights reveal that self-interacting proteins dictate the specificity, rewiring, and topological complexity of cellular signaling networks and macromolecular assemblies. Beyond their physiological roles, aberrant or dysregulated homotypic interactions disrupt cellular proteostasis, driving the formation of toxic non-native oligomers, pathological amyloid fibrillization, or aberrant liquid–liquid phase separation transitions linked to neurodegenerative and systemic diseases. This review provides a comprehensive overview of the state-of-the-art experimental methodologies, including proximity labeling, as well as the advanced computational frameworks, such as deep learning architectures and protein language models, used to map the structural dynamics of SIPs. Furthermore, we dissect the evolutionary trajectories of SIPs within protein–protein interaction networks, which are underpinned by dosage-balance constraints, and highlight their diverse functional advantages, ranging from allosteric modulation to biomolecular condensation. Finally, we summarize the molecular mechanisms linking pathological self-associations to human disorders, underscoring the emerging paradigm of targeting homotypic interfaces as a promising frontier for precision therapeutics.

## 1. Introduction

It is a foundational tenet of molecular biology that proteins rarely function in isolation; instead, they operate within intricate macromolecular networks to execute distinct cellular programs [1,2]. Within this interactome, protein self-interaction represents a specialized yet highly prevalent category of protein–protein interactions (PPIs), wherein two or more identical polypeptide chains physically associate to form symmetric homodimers and higher-order homo-oligomeric assemblies [3]. Self-interacting proteins (SIPs) constitute a substantial proportion of proteomes, with estimates ranging from 30 to 50% of all proteins capable of homotypic assembly in broad structural surveys [4] to more domain-specific predictions of ~45% for prokaryotic and ~20% for eukaryotic proteomes [5]. These insights demonstrate that homo-oligomerization is a dominant and biophysically robust evolutionary strategy rather than an anomalous event. Despite their prevalence, the absolute number of SIPs remains systematically underestimated in current interactome databases due to historical limitations in standard high-throughput PPI screening assays [6]. Furthermore, many homotypic interactions are inherently transient, dynamic, and context-dependent, presenting persistent challenges for conventional biochemical and biophysical characterization [7,8].

The evolutionary conservation of homomeric configurations is driven by their profound physicochemical and biological advantages over monomeric counterparts [9]. Homo-oligomerization optimizes mass-to-surface-area ratios, enhances thermodynamic stability, facilitates allosteric regulation, and provides precise spatial-temporal control over enzyme kinetics and cell signaling cascades [4]. At the system level, network biology has revealed that SIPs occupy highly centralized topological positions within empirical PPI networks [10]. They frequently act as principal network hubs and exhibit a significantly higher propensity for gene duplicability than non-SIPs [11]. This elevated duplicability is tightly constrained by whole-genome duplication events and dosage-balance mechanisms, suggesting that SIPs serve as critical evolutionary anchors that shape interactome complexity and modularity.

However, the structural plasticity that enables functional self-assembly also represents a pathological liability. When spatial-temporal regulation fails, or genetic mutations alter key interfacial residues, inappropriate or non-native protein self-associations can occur [9,12]. These dysregulated interactions often culminate in the formation of cytotoxic oligomers and insoluble macromolecular aggregates, precipitating proteostasis collapse and triggering severe human pathologies, including neurodegenerative disorders and systemic autoimmune syndromes. Consequently, a systematic, multi-scale understanding of protein self-interactions, bridging atomic-level structural interfaces with macro-level network topology, is essential for deciphering complex cellular processes and uncovering novel therapeutic vulnerabilities.

In this review, we comprehensively map the current landscape of SIP research. We begin by evaluating the latest experimental platforms and next-generation computational frameworks designed to detect and predict homotypic interfaces. We then analyze the evolutionary forces and structural drivers that dictate SIP duplicability and network centrality. Following this, we review the classical and emerging functional paradigms of homo-oligomerization, with particular emphasis on its role as a physical driver of liquid–liquid phase separation (LLPS). Finally, we discuss the direct causal links between aberrant protein self-interactions and human diseases, highlighting how uncovering these molecular mechanisms paves the way for innovative therapeutic modalities targeting homotypic interfaces.

## 2. Experimental Detection and Computational Prediction of Protein Self-Interactions

**Figure 1:**
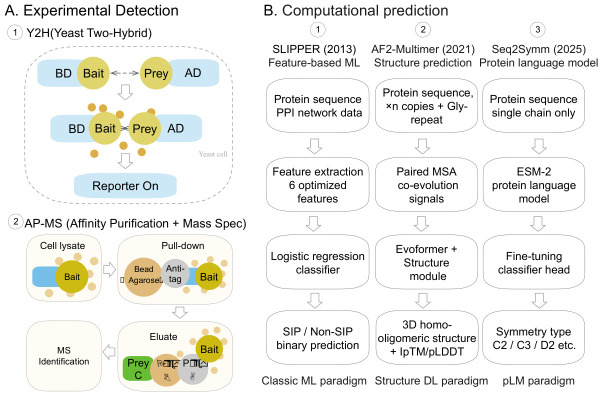
Representative experimental and computational approaches for the detection and prediction of protein self-interactions. Panel (**A**) illustrates the experimental methods for detecting SIPs, including yeast two-hybrid (Y2H) and affinity purification–mass spectrometry (AP-MS). Panel (**B**) presents three representative computational paradigms for SIP prediction: SLIPPER (feature-based machine learning with logistic regression), AlphaFold2-Multimer (deep learning-based structure prediction), and Seq2Symm (protein language model-based symmetry prediction).

As a specialized category of macromolecular crosstalk, protein self-interactions can be characterized through established experimental techniques and predicted via evolving computational frameworks originally developed for general PPIs (Figure 1).

### 2.1. Experimental Methods for Detecting Protein Self-Interactions

Currently, variants of yeast two-hybrid (Y2H) screening, single-step affinity purification coupled with mass spectrometry (AP-MS), and proximity labeling mass spectrometry (PL-MS) represent the most widely adopted high-throughput strategies for mapping PPI landscapes [13,14,15]. The classical Y2H system detects pairwise interactions based on the modular nature of eukaryotic transcription factors, wherein the DNA-binding domain and the activation domain can reconstitute a functional transcription factor when brought into proximity via bait–prey interaction [13,14]. To monitor self-interactions within this framework, identical protein species are expressed simultaneously as both bait and prey. To overcome the inherent limitations of the traditional Y2H assay, such as high false-positive rates, poor sensitivity to transient interactions, and incompatibility with hydrophobic membrane environments, several advanced variants have been engineered, including the mammalian-membrane two-hybrid assay (MaMTH) [15] and split-ubiquitin system [16].

To characterize stable or multi-subunit protein complexes, single-step AP-MS has emerged as a frontline high-throughput approach. By employing a single high-affinity epitope tag (e.g., FLAG or GFP), single-step AP-MS enables the rapid isolation of the target bait along with its associated homotypic or heterotypic partners under mild, near-physiological conditions [17,18]. Furthermore, to capture highly dynamic, transient, or spatially restricted interactions within living cells, proximity labeling techniques have become powerful complementary tools. This strategy utilizes a bait protein fused to an engineered enzyme (such as BioID, TurboID, or APEX) that covalently biotinylates neighboring prey proteins within a defined nanometer radius. The biotinylated proteome is subsequently enriched via streptavidin affinity purification and identified via mass spectrometry, effectively bypassing the stringent requirement of maintaining intact protein complexes during cell lysis [19,20,21].

### 2.2. Computational Approaches for Predicting Protein Self-Interactions

Despite advancements in experimental platforms, high-throughput assays still suffer from systematic biological artifacts and design limitations, rendering them less effective at comprehensively discerning homotypic self-interactions [6]. Moreover, conventional computational PPI prediction methods, such as phylogenetic profiling [22,23] and gene fusion-based strategies [24,25], are fundamentally unsuitable for the inference of SIPs. This limitation arises because the features critical for heterotypic PPI inference, such as genomic co-expression, co-localization, or distinct gene-fusion events, become either invariant or entirely uninformative when applied to identical protein partners. Consequently, SIPs are significantly underrepresented and underestimated in current interactome databases, which have catalyzed the development of tailored bioinformatics frameworks specifically designed for self-interaction prediction [26].

Liu et al. pioneered proteome-wide SIP prediction by developing SLIPPER (SeLf-Interacting Protein PrEdictoR) [6]. This framework employs a logistic regression model that integrates six optimized features distilled from an initial pool of eleven, capturing structural, functional, evolutionary, and network-topological attributes to differentiate between SIPs and non-SIPs. To expand prediction capabilities to proteins lacking network connectivity (which remained unaddressable by the topology-dependent SLIPPER model), the sequence-only web server SPAR (Self-interacting Protein Analysis serveR) was subsequently developed [27]. SPAR utilizes a Random Forest algorithm coupled with an enhanced sequence-encoding scheme that incorporates critical residue substitutions derived from fine-grained domain–domain interaction data [28].

The paradigm of structural biology and protein folding has recently been reshaped by deep learning, most notably exemplified by AlphaFold systems (e.g., AlphaFold2) [29]. Capitalizing on these architectures, modern machine learning models have significantly advanced sequence-based SIP prediction. For instance, Jia et al. developed NLPEI, a stacked autoencoder framework that fuses features extracted from natural language processing of amino acid sequences with evolutionary information from position-specific scoring matrices (PSSMs), achieving remarkable accuracies of 94.19% on human and 91.29% on yeast datasets [30]. Similarly, An et al. proposed RRN-SIFT, which integrates a recurrent neural network (RNN) with SIFT-extracted evolutionary features from PSSMs [31]. Although RRN-SIFT delivers exceptional accuracy (97.12% in humans and 94.34% in yeast), its widespread application is constrained by the heavy computational burden of PSSM generation and the inherent “black-box” opacity of RNN classifiers. It should be noted, however, that these high accuracy figures are heavily dependent on the specific benchmark datasets used, particularly the composition of negative samples, and may not generalize well across different species or experimental conditions due to inherent dataset redundancy [26].

To address the lack of consensus regarding the true comparative performance of these diverse models that were often masked by inconsistent datasets and heterogeneous evaluation protocols, Chen et al. systematically reviewed the field [26]. By establishing standardized, high-quality benchmark datasets and retraining nine representative predictors under a unified evaluation framework, they demonstrate that models combining evolutionary profiles (e.g., PSSMs) with deep learning architectures yield superior performance. However, poor cross-species generalization remains a critical bottleneck, providing a clear trajectory for future model refinement.

Concurrently, AI-driven strategies now enable large-scale, proteome-wide exploration of homotypic assemblies across the tree of life. For example, Schweke et al. developed a high-throughput computational pipeline that concatenates multiple protein copies via poly-glycine linkers to predict homo-oligomeric assemblies using AlphaFold2 across four representative species [5]. Their analysis of this structural landscape revealed that symmetric assemblies dominate the proteome, coiled-coil domains act as major drivers of quaternary-structure evolution in eukaryotes, and disease-associated mutations are significantly enriched at self-interacting interfaces.

Beyond prediction, the same computational principles—energy functions, sampling methods, and deep learning architectures—have been harnessed for the de novo design of self-interacting interfaces. This complementary field systematically constructs homotypic assemblies from scratch, enabling precise dissection of the biophysical determinants of self-interaction and providing a direct foundation for engineering therapeutic interface-targeting modalities. The recent review by Ranbhor et al. elegantly traces this evolution from physics-based energy functions and sampling toward AI-driven *de novo* design, offering a methodological framework that aligns closely with the computational approaches discussed here [32].

Building upon these foundations, the deep learning revolution has been further propelled by the introduction of AlphaFold3 [33]. Featuring a substantially updated diffusion-based architecture, AlphaFold3 expands prediction capabilities beyond static homotypic interfaces to accurately model full biomolecular assemblies, including interactions involving nucleic acids, small molecules, ions, and chemical modifications [33]. Crucially for SIP characterization, AlphaFold3 can generate multiple structural predictions that may reflect different conformational states within homo-oligomeric assemblies. Moreover, by leveraging its diffusion-based generative framework, AlphaFold3 can sample multiple distinct assembly configurations during inference, providing a principled avenue for probing alternative oligomeric topologies without the need for explicit multi-copy template construction. Nevertheless, predicting precise stoichiometric boundaries (e.g., accurately distinguishing an identical homotetrameric configuration from a homohexamer) directly from primary sequences without empirical constraints remains a recognized computational bottleneck [33].

To alleviate the heavy computational overhead required by downstream multi-copy structural predictions, alternative sequence-first strategies have been introduced. Specifically, Kshirsagar et al. fine-tuned pretrained protein language models (pLMs), such as ESM-2, to develop Seq2Symm [34]. This model rapidly and accurately predicts homo-oligomeric symmetry directly from single primary sequences, drastically reducing the computational overhead required by downstream tools like AlphaFold2-Multimer and enabling high-throughput structural annotation at the proteome scale. Nevertheless, predicting precise stoichiometric boundaries remains a recognized computational bottleneck, as the model’s symmetry predictions do not always explicitly encode the exact quaternary state. This difficulty stems from fundamental biophysical constraints: the same homotypic interface architecture can often accommodate multiple symmetric arrangements (e.g., cyclic C_4_ versus C_6_ symmetry) with comparable energetic favorability, and the co-evolutionary signals captured by pLMs primarily encode pairwise residue contacts that do not directly constrain higher-order topological assembly rules. Recent computational efforts are thus shifting from static quaternary structure prediction toward mapping the dynamic conformational landscapes and allosteric transitions of homotypic interfaces, which is pivotal for understanding transient signaling assemblies.

## 3. Roles of SIPs in PPI Network Evolution

**Figure 2:**
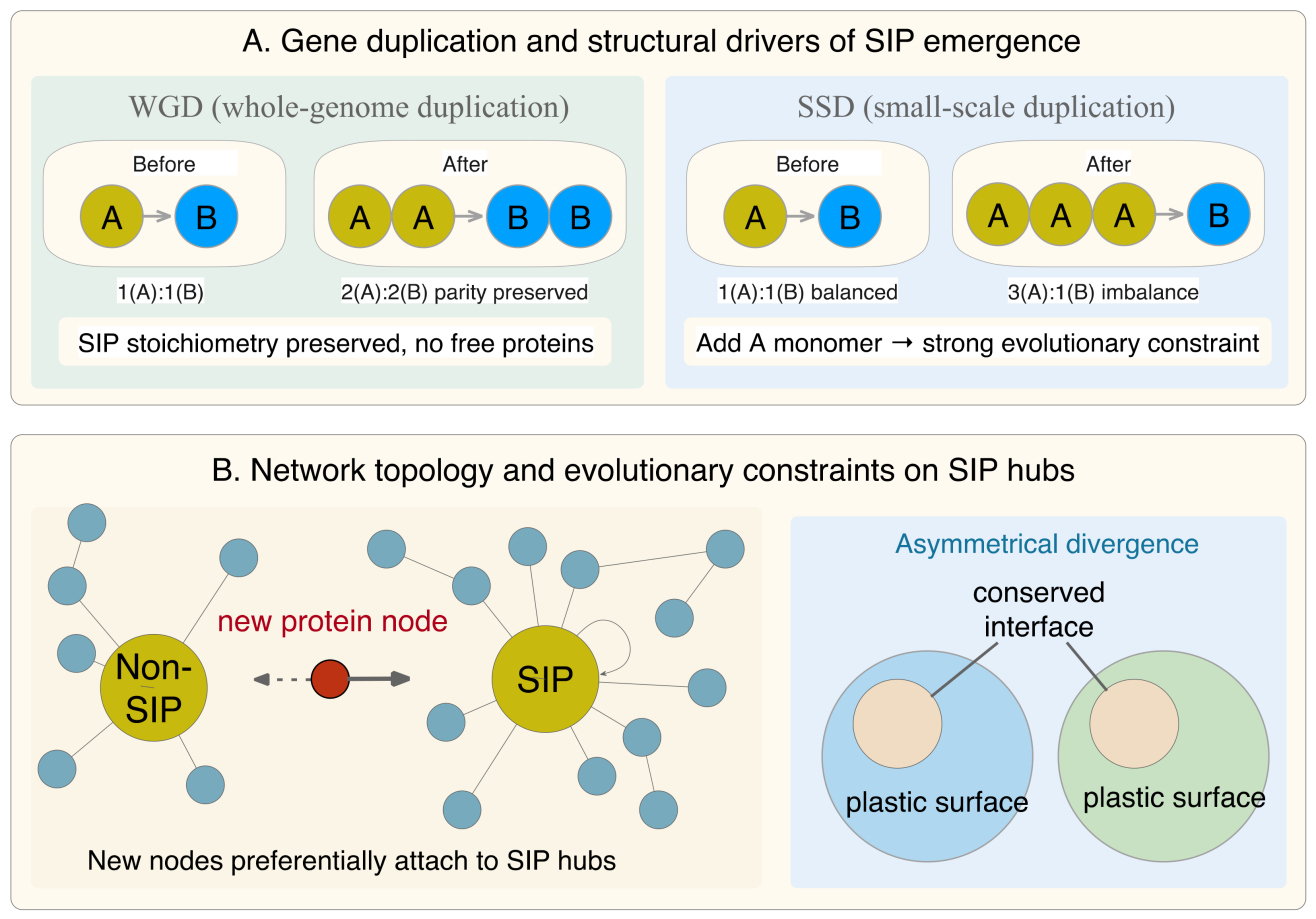
Roles of protein self-interactions in PPI network evolution. Panel (**A**): Gene duplication and structural drivers: Whole-genome duplication (WGD) preserves stoichiometric parity, whereas small-scale duplication (SSD) disrupts subunit ratios, leading to toxic aggregates (dosage-balance hypothesis). Panel (**B**): Network topology and evolutionary constraints: SIPs attract new protein nodes more readily than non-SIPs in PPI networks. SIP hubs exhibit asymmetrical divergence with conserved self-interacting interfaces and plastic solvent-exposed surfaces.

The birth, structural adaptation, and evolutionary dynamics of SIPs fundamentally shape the topology and modularity of PPI networks. By establishing homotypic molecular interfaces, SIPs serve as both critical toolkits for evolutionary innovation and anchoring structural units that dictate network complexity (Figure 2).

### 3.1. Evolutionary Mechanisms and Structural Drivers of Gene Duplicability in SIPs

Gene duplication serves as a primary engine for generating novel genetic material and driving interactive expansion across prokaryotic and eukaryotic genomes [5,6,35]. The classical duplication–divergence paradigm posits that following a duplication event, one gene copy retains its ancestral function under purifying selection, whereas the second copy is liberated from immediate selective pressure, accumulating mutations that facilitate either subfunctionalization or neofunctionalization [36,37,38,39,40].

Intriguingly, genes encoding SIPs exhibit a significantly higher propensity for duplicability compared to non-self-interacting counterparts [11]. Recent multi-omics analyses reveal that SIPs appear to have arisen far more frequently through whole-genome duplication rather than small-scale duplication [11]. This phenomenon is heavily underpinned by the dosage-balance hypothesis [37]. Because homo-oligomeric assemblies are highly sensitive to stoichiometric imbalances, an isolated duplication via small-scale duplication can disrupt the subunit ratio, leading to the formation of toxic non-native aggregates or dominant-negative phenotypes [37]. In contrast, whole-genome duplication simultaneously duplicates the entire interacting network, maintaining strict stoichiometric parity and thus permitting SIP duplicates to survive and diversify.

At the structural level, several distinct evolutionary mechanisms drive the initial emergence and subsequent stabilization of homotypic interfaces [3]. Over the past decade, structural biology paradigms have integrated macro-evolutionary models to detail these transitions. These transitions include:

- Exon shuffling, which rearranges structural modules to introduce symmetry [41];
- Formation of leucine zippers, providing canonical amphipathic alpha-helical coiled coils [42];
- Amino acid substitutions at surface residues that generate complementary hydrophobic patches [43];
- Targeted insertions/deletions (indels) within regions that modulate oligomeric states and optimize binding interfaces [44].

As highlighted by recent large-scale structuromic surveys utilizing AlphaFold2, symmetric assemblies dominate the quaternary landscape across the tree of life, with coiled-coil domains identified as paramount enablers of quaternary-structure evolution in eukaryotes [5]. These AI-driven structural insights reinforce that self-interaction represents an energetically favorable and biophysically robust pathway toward protein complexity.

### 3.2. Network Topological Centrality and the Evolutionary Constraints on Self-Interacting Hubs

Beyond individual gene fates, SIPs exert a profound influence on the macro-topology of biological networks. Within empirical interactomes, SIPs are significantly enriched and occur at a frequency far exceeding random expectation [37,45]. They function prominently as network hubs, possessing on average twice as many linking partners as non-SIPs [6,10]. Network growth dynamics indicate that the likelihood of a protein establishing new interactions over evolutionary timescales is proportional to its existing connectivity, a “rich-get-richer” preferential attachment mechanism heavily mediated by the structural plasticity of self-interactions [10]. A direct consequence of this topology is that numerous heterotypic protein complexes composed of paralogous dimers arise from the ancestral duplication and subsequent divergence of a single self-interacting homodimer [39].

This topological prominence directly addresses the long-standing evolutionary question: if SIPs act as critical hubs in the PPI network, are they more evolutionarily conserved? Cumulative bioinformatics and structural alignment analyses reveal that SIP hubs are indeed under intense evolutionary constraints and demonstrate exceptionally high sequence and structural conservation [38]. Because homotypic interfaces must maintain absolute structural symmetry to recognize identical copies of themselves, they are subject to stringent purifying selection to preserve function and prevent proteotoxic misfolding or deleterious non-native aggregation [9,12].

However, recent studies indicate that their molecular evolution typically follows a pattern of asymmetrical divergence [40]. Under this regime, the evolutionary strategy can be distilled into two distinct components (illustrated in the right part of panel B in Figure 2):

- Conserved homotypic core: The ancestral self-interacting interface remains structurally immobilized and highly conserved across orthologs. This rigidity safeguards the core homomeric configuration, which is essential for the SIP’s fundamental function.
- Plastic periphery: In contrast, the surrounding solvent-exposed surfaces and duplicated paralogs retain remarkable structural plasticity [38,40]. This flexibility allows for the accumulation of localized surface variations without compromising the central homotypic interaction.

This structural asymmetry provides a dual advantage: it allows SIP hubs to simultaneously preserve their essential homotypic functions while accumulating surface variations that can be wired into novel heterotypic pathways. Consequently, SIPs effectively balance evolutionary robustness with adaptive innovation, driving the progressive complexity of the interactome.

## 4. Functional Advantages of SIPs in Cellular Functions

### 4.1. Classical Functional Advantages of SIPs

**Figure 3:**
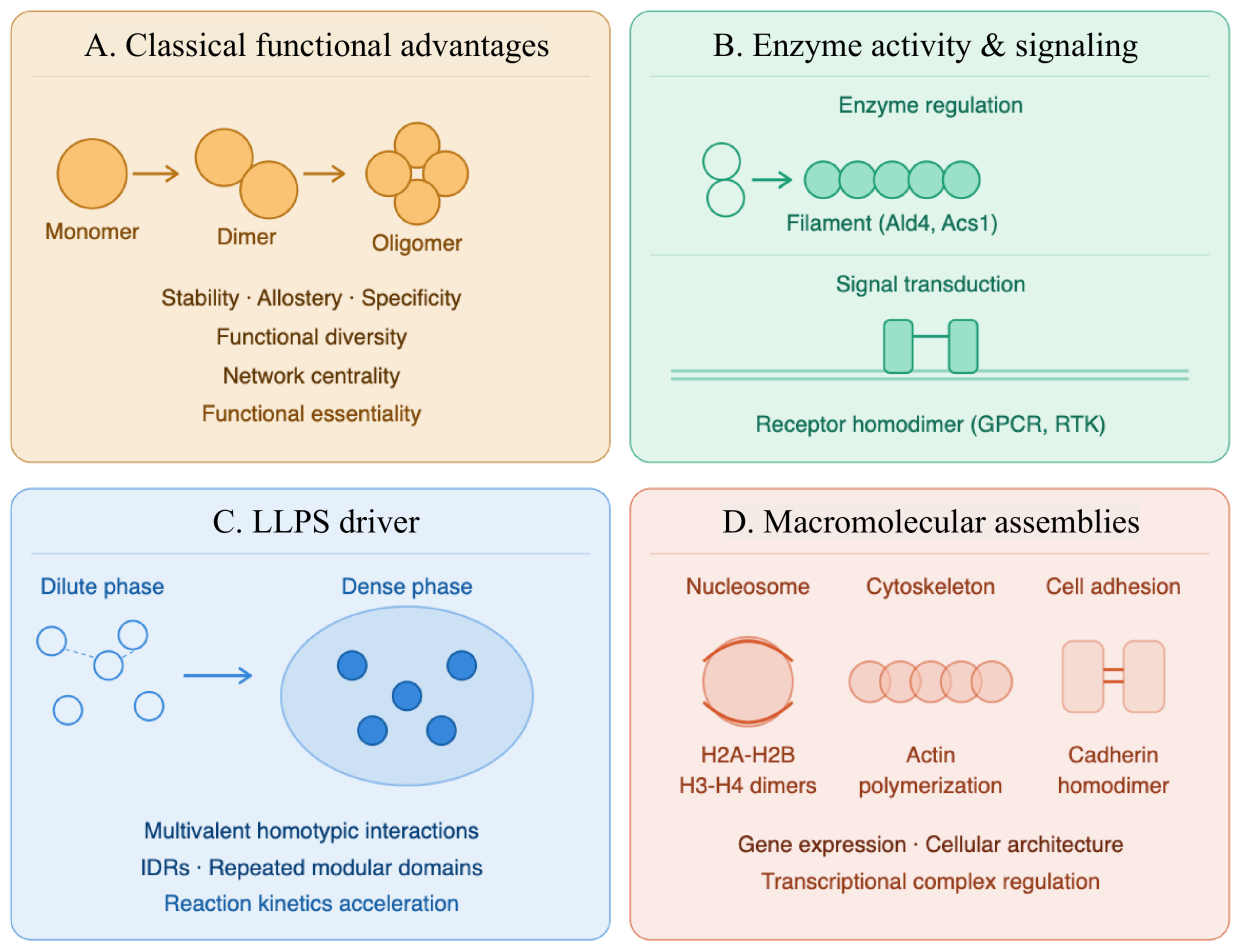
Functional advantages of protein self-interactions in cellular functions. Four panels illustrate: Panel (**A**). classical advantages including enhanced stability, allosteric regulation, greater structural complexity, network centrality and functional essentiality; Panel (**B**). regulation of enzyme activity and signal transduction—homotypic assembly modulates metabolic enzyme function (Ald4/Acs1 filaments) and GPCR signaling; Panel (**C**). Protein self-interactions as a physical driver of LLPS and biomolecular condensate formation; Panel (**D**). macromolecular assemblies including nucleosome, cytoskeleton, and cell adhesion rely on self-interactions.

Compared with monomers, SIPs, i.e., dimers and oligomers, have several distinct structural and functional advantages [9] (Figure 3A). It has been shown that self-interactions contribute to improved protein stability and control over the accessibility and specificity of active sites [4,9,35]. Via allostery, self-interactions play critical roles in the regulation of protein function [9]. Self-interactions can help proteins to form large structures and extend functional diversity without increasing genome size [3,46].

SIPs are found to have greater protein complexity than other protein counterparts, indicated by a larger number of functional protein domains [6]. SIPs are also revealed to be topologically more central in the protein interaction network [6,10] and consequently tend to be more likely to be functionally essential [6,11], which is in line with previous observations about the positive correlation between gene interaction network centrality and functional essentiality [47,48]. In addition, SIPs are detected to evolve at a statistically slower evolutionary rate (dN/dS) and have a much lower propensity to protein aggregation [6,49], indicating a stronger selection force acting on the evolution of SIPs.

### 4.2. Regulation of Enzyme Activity and Signal Transduction

Among SIPs, enzyme genes are significantly enriched, indicating that homotypic assembly plays a particularly prominent role in enzymatic function and regulation [6]; homodimers and homotetramers constitute the most highly represented quaternary architectures among these self-interacting enzymes, consistent with structural surveys across domains of life [5,35] (Figure 3B). While historical paradigms established that homotypic assembly modulates enzyme kinetics through classic cooperative allosteric transitions or localized active-site loop rearrangements, as briefly exemplified by hemoglobin and caspase-9 [50], recent state-of-the-art structural biology breakthroughs have shifted the research focus toward high-resolution visualization of dynamic macromolecular assemblies in vivo. A prominent milestone is the development of the “FilamentID” workflow combining cryo-electron tomography (cryo-ET) and single-particle cryo-EM, which demonstrated that conserved metabolic enzymes, such as mitochondrial aldehyde dehydrogenase (Ald4) and acetyl-CoA synthetase (Acs1), reversibly polymerize into elaborate homo-oligomeric filamentous bundles to suppress or switch metabolic activity during cellular dormancy and nutrient starvation [51]. In these filamentous assemblies, the active sites of individual enzyme subunits become sterically occluded or allosterically locked into an inactive conformation, thereby directly blocking substrate access and catalytic turnover. Furthermore, recent cryo-EM heterogeneity analyses of soluble angiotensin-converting enzyme (ACE) homodimers have revealed that stable homotypic interfaces tightly coordinate independent opening–closing dynamics of individual catalytic pockets, presenting an alternative blueprint for domain-specific allosteric drug design [52].

Beyond metabolic catalysis, self-interaction represents an absolute spatial gatekeeper for transmembrane signal transduction [53]. A diverse array of cell-surface receptors historically known to rely on oligomerization, including G-protein-coupled receptors (GPCRs) [54,55,56], receptor tyrosine kinases [57], and cytokine receptors [58], are now being mapped at atomic resolutions to capture transient, asymmetric signaling states rather than static homomeric configurations. For instance, recent cryo-EM structures of the visual system-specific orphan Class C GPCR, GPR179, revealed that its transmembrane domain forms a homodimer via a noncanonical TM1/7 interface secured by a single inter-protomer disulfide bond. This establishes a specialized geometric architecture optimized for the highly curved membranes of retinal dendritic tips [59]. Similarly, high-resolution structures of active Class A GPCR homodimers, such as the apelin receptor complexed with endogenous agonists, demonstrate that ligand occupancy can drive highly asymmetric structural reorganizations across the homotypic interface, allowing a single protomer to selectively steer downstream heterotrimeric G-protein coupling or biased signaling pathways [60]. Collectively, these recent structural insights reinforce that protein self-interaction is not merely a mechanism for static molecular stabilization, but a highly tunable, multi-state regulatory axis that governs cellular responsiveness.

### 4.3. Protein Self-Interactions as a Physical Driver of LLPS

Beyond the assembly of rigid, discrete macromolecular complexes, protein self-interactions serve as a primary physical and biophysical driver for LLPS and the formation of membraneless biomolecular condensates [61] (Figure 3C). This spontaneous demixing process occurs when multivalent homotypic interactions cross a critical thermodynamic threshold, enabling identical protein copies to segregate from a homogeneous mixture into a highly concentrated dense phase that dynamically coexists with the dilute solution [61,62]. SIPs that drive cellular LLPS are typically enriched with repeated modular binding domains or intrinsically disordered regions (IDRs), which facilitate weak, transient, and highly cooperative intermolecular crosstalk [61].

This self-interaction-driven compartmentalization yields profound functional advantages: it allows cells to rapidly sequester macromolecules, establish specialized microenvironments without lipid membranes, and dynamically modulate signal transduction cascades [61,63]. By locally concentrating specific enzymes and their cognate substrates within these homotypic condensates, reaction kinetics can be accelerated by orders of magnitude, effectively controlling complex cellular programs under shifting physiological conditions [63].

### 4.4. Macromolecular Assemblies in Gene Expression and Cellular Architecture

Protein self-interactions are critical for the assembly of protein complexes involved in gene expression (Figure 3D). In both prokaryotic and eukaryotic organisms, the transitions of the oligomerization status of transcriptional protein complexes are known to be essential to transcriptional state control and consequently the survival of the organisms [64]. A premier structural manifestation of self-interaction in genetic regulation is the assembly of the eukaryotic nucleosome core, which relies on the highly coordinated homotypic and heterotypic dimerization of histone dimers (H2A-H2B and H3-H4) [65,66]. In addition, the functional importance of protein self-interactions has also been reported in other crucial biological processes, such as the assembly of the cytoskeleton [67] and cell–cell adhesion processes [68,69].

## 5. Protein Self-Interactions and Diseases

**Figure 4:**
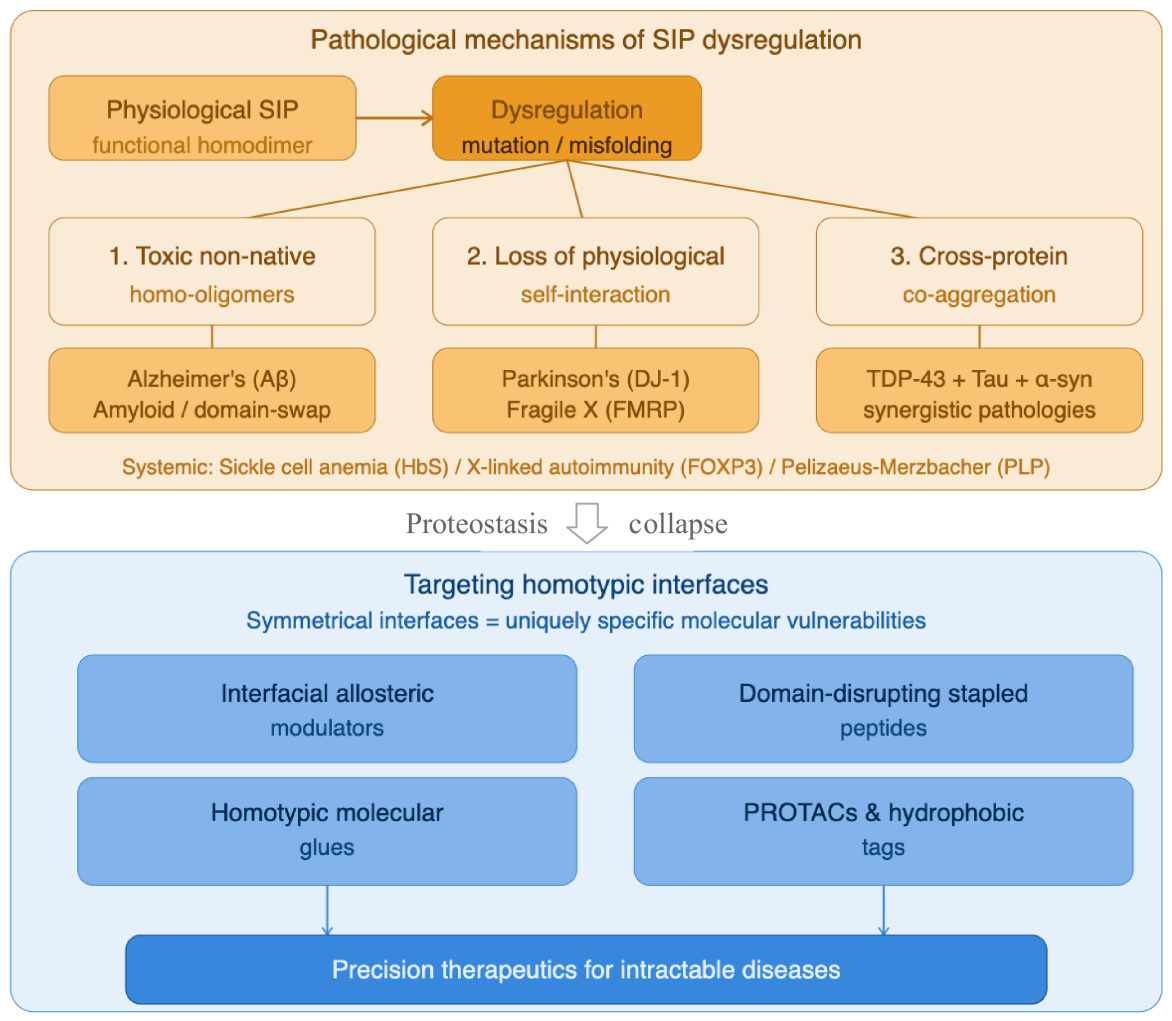
Protein self-interactions in disease and therapeutic targeting. The upper panel illustrates three disease mechanisms: (1) toxic non-native homo-oligomers (amyloid-beta fibrillization and domain-swapped assemblies in Alzheimer’s disease); (2) loss of physiological SIP (DJ-1 dimer disruption in Parkinson’s disease, FMRP oligomerization impairment in fragile X syndrome); (3) cross-protein co-aggregation (TDP-43 co-aggregating with Tau and α-synuclein). The systemic section lists systemic diseases: sickle cell anemia (HbS polymerization), autoimmunity (FOXP3 dimer disruption), and PLP premature oligomerization in Pelizaeus–Merzbacher disease [76]. The lower panel presents four therapeutic strategies targeting homotypic interfaces: interfacial allosteric modulators, domain-disrupting stapled peptides, homotypic molecular glues, and PROTACs/hydrophobic tags.

Given that SIPs play a critical role in regulating cellular functions, a substantial evolutionary selection pressure operates to preserve their native quaternary structures, notably driving a powerful negative selection force against aberrant protein aggregation [49]. Nevertheless, dysregulated or non-native self-associations frequently exert highly deleterious biological effects (Figure 4). These pathological outcomes typically arise from either the formation of toxic, non-native homo-oligomers or the structural impediment of physiological homomer assembly, both of which can fundamentally disrupt cellular proteostasis and culminate in the pathogenesis of various human disorders, most notably neurodegenerative diseases [70,71,72].

A classic paradigm of inappropriate protein self-association is the formation of amyloid fibrils, a process often accompanied by the production of aberrant, domain-swapped protein homo-oligomers [73]. Historically, this fibrillization cascade was first recognized as a hallmark pathogenic feature of Alzheimer’s disease, a progressive neurodegenerative disorder characterized by widespread amyloid deposition in the cerebral neuropil and vasculature. Accumulating evidence highlights that the self-assembly of soluble amyloid-beta peptides into cytotoxic oligomers and cross-beta-sheet-structured fibrils triggers synaptic loss, neuroinflammation, and subsequent neuronal death, firmly linking this self-interaction process to Alzheimer’s disease progression [71,72,74,75]. Beyond single-protein self-association, pathological aggregation can extend to cross-protein co-aggregation, where a SIP aberrantly interacts with other pathogenic proteins. For instance, TDP-43, which normally functions as a homodimer, can co-aggregate with Tau, α-synuclein, and other disease-related proteins, synergistically exacerbating neurodegenerative pathologies across multiple disease boundaries [72].

Beyond structural misfolding, temporal dysregulation of homo-oligomerization can be equally detrimental. For instance, the premature oligomerization of proteolipid protein within the endoplasmic reticulum of oligodendrocytes disrupts vesicular trafficking and induces chronic ER stress, contributing significantly to the demyelinating pathology of Pelizaeus–Merzbacher disease [76]. Conversely, the loss or failure of physiological protein self-interaction is a potent driver of disease. A prominent example occurs in early-onset familial Parkinson’s disease, where causative missense mutations (such as L166P or M26I) disrupt the critical homodimerization interface of the multifunctional protein DJ-1 [77]. This dimerization failure destabilizes the protein, rendering it susceptible to rapid proteasomal degradation and compromising its neuroprotective capacity against oxidative stress. Similarly, point mutations in the fragile X mental retardation protein (FMRP) compromise its structural stability and self-assembly capacity, undermining both its protein integrity and RNA-binding regulation, thereby underlying the molecular pathophysiology of fragile X syndrome [78].

Pathological protein self-interactions extend beyond neurological conditions to systemic diseases. For instance, specific point mutations in the hemoglobin gene alter the conformation of its deoxygenated form, driving its pathological polymerization into rigid fibers that distort erythrocytes and cause sickle cell anemia [79]. Furthermore, disrupting the domain-swapped homodimerization of the forkhead transcription factor FOXP3, or its regulatory hetero-oligomerization with FOXP1, profoundly impairs its DNA-binding efficiency and transcriptional suppressor properties. This molecular breakdown compromises regulatory T-cell function, leading to the severe multi-organ autoimmunity characteristic of X-linked autoimmunity and allergic dysregulation syndrome [80].

## 6. Conclusions and Future Perspectives

Over the past decade, our understanding of SIPs has undergone a profound paradigm shift. Once viewed as isolated biochemical phenomena, homodimers and higher-order homo-oligomers are now recognized as foundational architectural pillars and regulatory hubs that dictate the topological complexity, modularity, and evolutionary dynamics of the cellular interactome. The recent notable convergence of high-resolution in vivo experimental techniques, with transformative, deep-learning-driven structural biology frameworks and advanced protein language models, has fundamentally changed our ability to map homotypic interfaces at a proteome-wide scale.

Despite these significant strides, several critical bottlenecks remain to be resolved in future research. A primary challenge lies in shifting from the prediction of static quaternary structures toward decoding the highly dynamic, transient, and context-dependent conformational landscapes of low-affinity SIPs within their crowded native cellular environments. Computationally, predicting precise stoichiometric boundaries and structural symmetry directly from primary sequence data without prior empirical constraints remains an open hurdle. Few sequence-derived features are sufficient to disambiguate alternative symmetry states whose free-energy differences can be vanishingly small, and no current deep learning architecture autonomously infers the correct oligomeric number from sequence information alone. Furthermore, as the field of biomolecular condensation expands, a vital trajectory for biophysical research will be to elucidate the exact thermodynamic and molecular thresholds that govern how homotypic SIPs transition from functional, liquid-like phase-separated states into pathological, irreversible solid aggregates.

Looking forward, the most compelling translational impact of SIP research resides in the realm of precision therapeutics. Because the symmetrical topographies of homotypic interfaces are highly distinct from those of heterotypic complexes, they offer uniquely specific, historically untargeted molecular vulnerabilities. Emerging pharmacological strategies are rapidly shifting away from classical active-site inhibition toward the rational design of interfacial allosteric modulators, domain-disrupting stapled peptides, and homotypic molecular glues. Interfacial allosteric modulators bind to a site distinct from the active site to stabilize a specific symmetric or asymmetric conformational state, thereby indirectly modulating homo-oligomer function. By contrast, molecular glues are designed to directly induce or stabilize a specific protein–protein interaction at the homotypic interface, often promoting the formation of a functional or degradable complex that would not naturally occur. Moreover, leveraging targeted protein degradation modalities, such as designing PROTACs or hydrophobic tags that selectively recognize and eliminate cytotoxic, misfolded homo-oligomers while sparing their native, physiological counterparts, presents a revolutionary frontier. Ultimately, unraveling the precise regulatory mechanisms of protein self-interaction will not only deepen our fundamental grasp of cellular evolutionary biology but will also unlock innovative therapeutic strategies to combat currently intractable neurodegenerative, oncogenic, and autoimmune disorders.

## Data Availability

No new data were created or analyzed in this study. Data sharing is not applicable to this article.

## Abbreviations

The following abbreviations are used in this manuscript:

PPIs	Protein–protein interactions
SIPs	Self-interacting proteins
LLPS	Liquid–liquid phase separation
Y2H	Yeast two-hybrid
AP-MS	Affinity purification–mass spectrometry
